# Distinct mechanisms subserve location- and object-based visual attention

**DOI:** 10.3389/fpsyg.2014.00456

**Published:** 2014-05-21

**Authors:** Wei-Lun Chou, Su-Ling Yeh, Chien-Chung Chen

**Affiliations:** ^1^Department of Psychology, National Taiwan UniversityTaipei, Taiwan; ^2^Department of Psychology, Fo Guang UniversityYilan, Taiwan; ^3^Neurobiology and Cognitive Science Center, National Taiwan UniversityTaipei, Taiwan

**Keywords:** attention mechanisms, location-based attention, object-based attention, threshold versus external noise contrast (TvC) function, noise-masking paradigm, divisive inhibition model

## Abstract

Visual attention can be allocated to either a location or an object, named location- or object-based attention, respectively. Despite the burgeoning evidence in support of the existence of two kinds of attention, little is known about their underlying mechanisms in terms of whether they are achieved by enhancing signal strength or excluding external noises. We adopted the noise-masking paradigm in conjunction with the double-rectangle method to probe the mechanisms of location-based attention and object-based attention. Two rectangles were shown, and one end of one rectangle was cued, followed by the target appearing at (a) the cued location; (b) the uncued end of the cued rectangle; and (c) the equal-distant end of the uncued rectangle. Observers were required to detect the target that was superimposed at different levels of noise contrast. We explored how attention affects performance by assessing the* threshold versus external noise contrast (TvC) functions* and fitted them with a divisive inhibition model. Results show that location-based attention – lower threshold at cued location than at uncued location – was observed at all noise levels, a signature of signal enhancement. However, object-based attention – lower threshold at the uncued end of the *cued* than at the *uncued* rectangle – was found only in high-noise conditions, a signature of noise exclusion. Findings here shed a new insight into the current theories of object-based attention.

Our visual world is full of information; however, not all can be selected for further processing due to limited capacity. Mechanisms of attention are thus employed to prioritize the processing of particular information. Past studies have shown that visual attention can be allocated either to a spatial location or to an object, called location-based attention or object-based attention, respectively (Posner, 1980; Duncan, 1984; Tipper et al., 1991; Egly et al., 1994; Gibson and Egeth, 1994; Brawn and Snowden, 2000).

In a seminal work, Egly et al. (1994) used a double-rectangle display to demonstrate both location-based attention and object-based attention. They presented two outlined rectangles, with one end of one rectangle brightened as a cue to indicate the possible location of a target. The target was a small solid square, shown subsequently within one end of a rectangle. Location-based attention was indicated by the* spatial-cueing* effect: reaction times (RTs) were shorter when the target appeared at the cued location than the uncued location. Object-based attention was indicated by the *same-object advantage*: RTs were shorter when the target appeared at the uncued end of the *cued* rectangle than at the *uncued* rectangle, with an equal cue-to-target distance between the two. Concurring with Egly et al. (1994), a series of studies using various stimuli and tasks have demonstrated the spatial-cueing effect and the same-object advantage (Moore et al., 1998; Abrams and Law, 2000; Lamy and Tsal, 2000; Moore and Fulton, 2005; Brown et al., 2006; Matsukura and Vecera, 2006; Shomstein and Behrmann, 2008).

The spatial-cueing effect has been explained by the movement of attention from one location to another in visual space. On valid trials, a shift of attention can be initiated to the expected target *location* before the target appears, thereby producing an RT or accuracy benefit (Posner, 1980). On the two kinds of invalid trials, however, a shift of attention would be initiated to a location on the wrong site of the display from the actual target location. This would produce an RT or accuracy cost because attention would need to be realigned with the correct target location after the target’s appearance.

The same-object advantage has been explained mainly by two competing theories. The *spreading hypothesis* states that when attention is cued to a location within an object, attention will spread automatically from the cued location to the whole object (e.g., Davis and Driver, 1997; Kasai and Kondo, 1997; Richard et al., 2008). Such spread of attention explains the participants’ better visual performance when the target was shown on the cued object than on the uncued object. Since the attentional modulation is triggered by a location cue and spreads to the whole object, the same-object advantage should be an instance of location-based attention. That is, the underlying mechanism of object-based attention is the same as that of location-based attention. In addition, it is shown that improvement of visual performance in a location-based attention task can be due to (a) the participant being more sensitive to a target at the cued location than that at the uncued one; and/or (b) the participant being less influenced by irrelevant visual information (Lu and Dosher, 1998). Hence, these two factors should be able to account for object-based attention as well, if it shares the same mechanism as location-based attention.

On the other hand, the *prioritization hypothesis* (Shomstein and Yantis, 2002) suggests that object-based attention reflects a specific attentional prioritization strategy rather than the modulation of an early sensory enhancement extending from the location-based attention. That is, the prioritization hypothesis does not take any position regarding the similarity of the mechanisms between location- and object-based attention. At best, it would predict different mechanisms for the *exogenous* spatial-cueing effect and the strategically object-based scanning strategy. Therefore, the same-object advantage cannot be explained by a change in early sensory mechanisms.

Here, we are interested in the mechanisms that subserve location- and object-based attention, especially whether the mechanisms underlying these two types of attention are the same. Notice that previous investigations adopting the double-rectangle method generally used RT measurement with a single level of task difficulty (Egly et al., 1994; Moore et al., 1998; Abrams and Law, 2000; Lamy and Tsal, 2000; Moore and Fulton, 2005; Brown et al., 2006; Shomstein and Behrmann, 2008). RT measurement may reflect processing speed, response bias, or a combination of the two (Ratcliff, 1978), making it hard to infer the underlying mechanisms. In addition, while an estimation of response variability is important to evaluate certain theories of location-based attention (Lu and Dosher, 1998), it is difficult to separate measurement error from the experimental procedure and the variability of the internal responses in the RT measurement.

We used a noise-masking paradigm (Nagaraja, 1964; Legge et al., 1987; Pelli, 1991; Lu and Dosher, 1998) that can evaluate the variability in the response of the visual system in the double-rectangle display to probe the mechanism(s) of location-based attention and object-based attention. In a typical noise-masking paradigm, the task of the observer is to detect a pre-designated target that is superimposed on a patch of white noise. In the context of our experiment, the target was a periodic pattern defined by a Gabor function, which is a product of a sine wave and a Gaussian envelope, while the noise was a random modulation of luminance. The intensity of the noise mask was defined by contrast, or the theoretical half range of the luminance modulation defined by a uniform distribution divided by the mean luminance. By systematically measuring the target threshold at different external noise levels, we can measure *the threshold versus external noise contrast (TvC) functions*. With an appropriate model, this information allows an estimation of the response properties and variability of the target detection mechanisms, thus providing a more comprehensive estimation of various perceptual mechanisms (Nagaraja, 1964; Legge et al., 1987; Pelli, 1991; Lu and Dosher, 1998; Chen and Tyler, 2001; Wu and Chen, 2010).

By taking advantage of the double-rectangle method, we evaluated the TvC functions of attended and unattended location/object within a single paradigm. In a two-alternative intervals choice task (**Figure 1**), participants were asked to detect a Gabor target that was superimposed on a noise pattern. The displays, if not stated otherwise, consisted of two vertical rectangles that were presented on each side of fixation. The four ends of the rectangles were where the cue (or target) was likely to occur. The target could occur at one of the three possible locations: the cued location (*valid*), the uncued location but on the cued object (*same-object*), or an equidistant location on the uncued object (*different-object*). Then, we measured the TvC functions for all the different conditions so that we can compare location-based attention and object-based attention and infer their mechanisms directly. If their mechanisms are identical, they should show the same kind of shift in the TvC functions.

**FIGURE 1:**
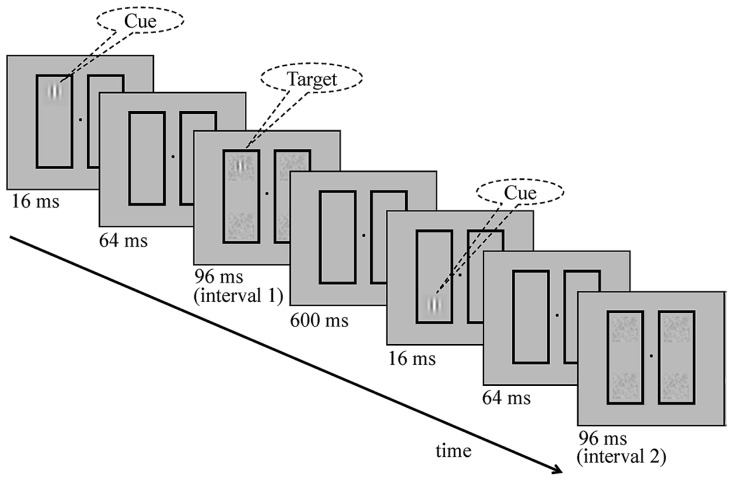
**Schematic overview of a typical valid trial with the target showing in interval 1.** The task was to detect the target (a Gabor patch) superimposed on different levels of noise (mask) contrast in a two-alternative forced-choice paradigm. In each interval, a cue was flashed first for 16 ms, followed by a 64 ms blank, and then a stimulus presentation (either target-plus-noise mask or noise mask alone). Two intervals were separated by a 600 ms blank. The rectangles and the fixation point were always on-screen.

## MATERIALS AND METHODS

### ETHICS STATEMENT

The use of human participants was approved by the IRB of National Taiwan University Hospital and followed the guideline of Helsinki Declaration. The written informed consent was obtained from each participant.

### APPARATUS

Two ViewSonic (15′′) CRT monitors, each driven by a Radeon 7200 graphic board, were used to present the stimuli. The graphic board provided 10-bit digital-to-analog converter depth and was controlled by a Macintosh computer. A beam splitter was used to combine lights from the two CRT monitors. The target was presented on one monitor and the cue and the external noise patch (mask) on the other. This two-monitor setup had the advantage that the contrast of the target could be controlled independently while keeping the context (the cue and the mask) identical in two intervals of a trial. At a viewing distance of 128 cm, the resolution on a 640 × 480 pixels monitor was 60 pixels per degree. The refresh rate of the monitors was 66 Hz. The viewing field was 10.7° × 8° (horizontal × vertical), and the mean luminance of the displays was 74.9 cd/m^2^. The LightMouse photometer (Tyler and McBride, 1997) was used to measure the full-detailed input-output intensity function of the monitors, and this information was then used to compute linear lookup table settings so as to linearize the output within 0.2%.

### STIMULI AND DISPLAY

**Figure 1** illustrates the stimuli and sequence of events for a trial. The displays are comprised of a pair of adjacent vertical rectangles. The fixation was a small dot. Each rectangle (1.63° × 4.88°, with a stroke width 0.13°) was centered 3° from fixation. The cue and the target were vertical Gabor patches defined by the following equation:

$$G\left(x,y,c,u_{x},u_{y}\right)=L+L*c*\cos(2\pi fx)*\exp\left(-\frac{{\left(x-u_{x}\right)}^{2}}{2\sigma^{2}}\right)*\exp\left(-\frac{{\left(y-u_{y}\right)}^{2}}{2\sigma^{2}}\right)\prime$$

where *L* was the mean luminance, *c* was the contrast ranging from 0 to 1, *f* was the spatial frequency, σ was the scale parameter of the Gaussian envelope, *u*_x_ was the horizontal displacement, and *u*_y_ was the vertical displacement. Both Gabor patches had a spatial frequency (*f*) of 1.3 cycles/deg and a scale parameter (σ) of 0.3536°. The contrast of the cue (*c*) was -6 dB or 50%. For each external noise frame the pixel gray-levels were sampled from a Gaussian distribution.

### PROCEDURE

A two-alternative forced-choice paradigm was used to measure the threshold of the target (**Figure 1**). The cue was presented at one of four possible locations in each interval. After that, the target was presented at one of the three possible locations: (1) the cued location (*valid* trials), (2) the uncued end within the cued object (*same-object* trials), or (3) the uncued end within the uncued object (*different-object* trials) in one of the intervals.

A fixation display (a central fixation point and two outline rectangles) was presented first, followed by a 16-ms cue display, then a 64-ms fixation display, and finally a 96-ms target display (a target and four mask patches). The stimulus onset asynchrony between the cue and the target was 80 ms, the inter-stimulus-interval within a trial was 600 ms, and the inter-trial-interval was 800 ms. At the beginning of each trial an audio tone was presented as a signal to start. Correct and incorrect responses were followed by auditory feedbacks.

Each block of seven external noise levels (-∞,-26, -22, -18, -14, -10, -6 dB) were presented in random order, and each block contained the three attention conditions (valid, same-object, and different-object). The threshold was defined at 75% correct response level, measured by the PSI threshold-seeking algorithm (Kontsevich and Tyler, 1999). For each threshold measurement, two practice trials preceded 40 formal trials. Within a single block, four thresholds were measured in an interleaved way – two for the valid condition, one for the same-object and one for the different-object conditions, making the total number of valid trials (84 trials) twice as many as that of the same-object or different-object trials (42 trials). That is, the cue validity for predicting the target location was 50%. The sequence of trials was pseudo-randomized. The TvC function of the valid condition is the average of two threshold measurements. Each data point reported was an average of four to eight repeated measures. The task was to indicate which interval contained the target by pressing a corresponding key. Participants were told that the two outline rectangles were task-irrelevant, and they were well informed about the cue-target relationship.

### PARTICIPANTS

Three participants with normal or corrected-to-normal visual acuity were tested. RY and TH were naïve as to the purposes of this study and WL was one of the authors.

## RESULTS

**FIGURE 2:**
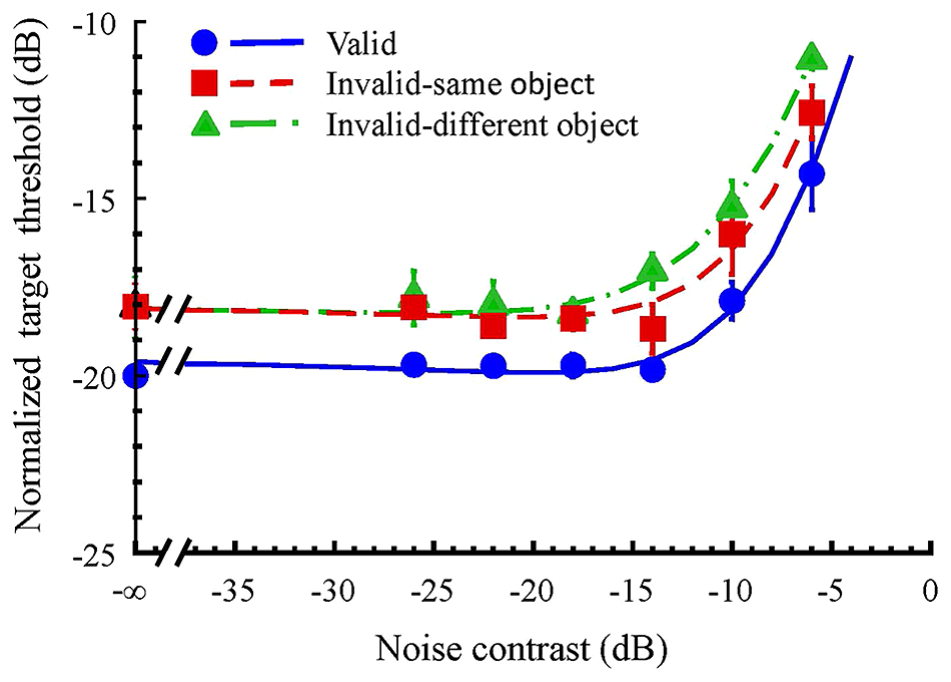
**Target threshold versus noise contrast functions.** Each data point represents the average of the normalized threshold from three observers. The blue circles and the solid curve denote the TvC function for the valid condition; the red squares and the dashed curve, the same-object condition; the green triangles and the dash-dot curve, the different-object condition. The smooth curves are fits of the model discussed in the text. The error bars are the estimated one standard error of normalized individual difference.

**Figure 2** shows the result averaged across three participants. The blue circles and solid curve denote the TvC function for the valid condition; red squares and dash curve, the same-object condition; and green triangles and dash-dot curve, the different-object condition. To account for the individual difference in overall sensitivity to the target, we scaled each threshold by that measured at zero noise contrast of the valid condition of the corresponding participant before averaging. When there was no noise mask, the threshold for the valid condition was lower than that for both invalid conditions. The difference was 2 dB [*t*(2) = 3.46, *p* = 0.037 < 0.05] between the valid cue and both the invalid conditions. Such difference between the valid and invalid conditions remained as the mask increased. Thus, the TvC functions of the invalid conditions look like a vertically shifted copy of the valid condition on log–log coordinates. Such general facilitation on target detection suggests that the effect of the valid cue was to increase the sensitivity to the target (Cohn and Lasley, 1974; Lu and Dosher, 1998; Zenger et al., 2000; Pestilli and Carrasco, 2005; Chen and Tyler, 2010).

The target detection thresholds were not influenced by the low contrast noise mask for all attention conditions. As a result, all TvC functions were flat at low noise contrasts. When the noise contrast reached a critical value, the threshold began to increase with noise contrast. Here, whether or not the cue and the target were within the boundary of an object had an effect. The threshold increment for the different-object condition started at a lower noise contrast than that for the same-object condition. As a result, the TvC function for the different-object condition showed a leftward shift from the TvC function for the same-object condition. This suggests that the noise effect on target detection in the same-object condition is different from that in the different-object condition.

Our result cannot be explained by an inter-hemispherical effect. In a control condition, we used horizontal rectangles as the objects. We measured the target threshold at noise level -∞ and -6 dB. There was no statistical significant difference [*t*(11) = -1.1, *p* = 0.30] in target threshold between the vertical and the horizontal object configurations, averaged across all conditions and observers.

## MODEL

**FIGURE 3:**
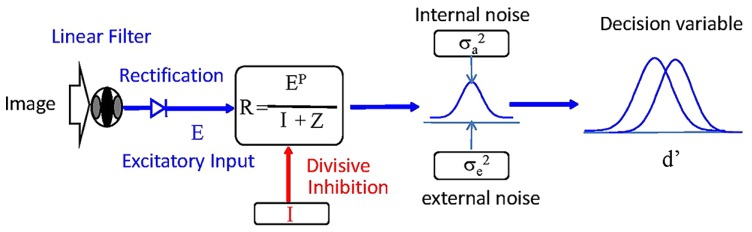
**Diagram of the model used to fit the data.** See text for details.

We fitted the TvC functions by a version of the divisive inhibition model (Ross and Speed, 1991; Wilson and Humanski, 1993; Foley, 1994; Teo and Heeger, 1994; Watson and Solomon, 1997; Snowden and Hammett, 1998; Chen and Foley, 2004) modified to account for the noise-masking experiment (Lu and Dosher, 1998; Goris et al., 2008; Chen and Tyler, 2010). This model integrates features from the divisive inhibition models for pattern detection and discrimination (Foley, 1994; Chen and Foley, 2004) and conventional models for noise masking (e.g., Lu and Dosher, 1998). Chen and Tyler (2010) used a similar model to account for the cueing effect in a noise-masking paradigm. **Figure 3** shows a diagram of this model. There are several stages in this model. The first stage is a band of linear filters operating on the input images. The excitation of a linear filter is then half-wave rectified, raised to a power and scaled by a divisive inhibition input to form the response of the target detector. The decision variable is the ratio of the response of the target detector and the noise from different sources.

Each mechanism *j* contains a linear operator within a spatial sensitivity profile *f*_j_(*x*,*y*). The excitation of this linear operator to the *i*-th image component *g*_i_(*x*,*y*) is specified as:

(1) $$E_{{ij}^{\prime }}=\sum_{x}\sum_{y}f_{j}\left(x,y\right)g_{i}\left(x,y\right)$$

where the linear filter *f*_j_(*x*,*y*) is defined by a Gabor function (see “Materials and Methods”). Suppose that the image component *g*_i_(*x*,*y*) has a contrast *C*_i_. Summing over *x* and *y*, Eq. (1) can be simplified to

(1′) $$E_{{ji}^{\prime }}={Se}_{ji}C_{i\,}\,$$

where Se_ji_ is a constant defining the excitatory sensitivity of the mechanism to the stimulus (*j* = *t* for the target and *j* = *m* for the mask). Detailed derivation of Eq. (1)’ from Eq. (1) has been discussed elsewhere (Chen and Tyler, 1999; Chen et al., 2000).

The excitation of the linear operator is half-wave rectified (Foley, 1994; Teo and Heeger, 1994; Foley and Chen, 1999) to produce the rectified excitation *E*_ji_

(2) $$E_{ji}=\max\left(E_{{ji}^{\prime }},0\right)$$

where max denotes the operation of choosing the greater of the two numbers.

The total excitation of the *j*-th mechanism *E*_j_ is the sum of excitations produced by all image components. The response of the *j*-th detector is then *E*_j_, raised by a power *p* and divided by a divisive inhibition term *I*_j_ plus an additive constant *z*. That is,

(3) $$R_{j}=E_{j}^{p}/\left(I_{j}+z\right)$$

where *I*_j_ is the summation of a non-linear combination of the excitations of all relevant mechanisms. This divisive inhibition term *I*_j_ can be represented as

(4) $$I_{j}=\sum_{i}{\left({Si}_{j,i}C_{i}\right)}^{q}\,$$

where *Si*_j,i_ is the weight of the contribution from each component to the inhibition term.

The contribution of a detector to the visual performance is limited by the noise. We consider two sources of noise in this model: the internal noise inherent in the system, and the external noise provided by the noise patterns. The variability produced by the internal noise, $\sigma_{a}^{2}$ , is a constant for all detectors in the model. The variability produced by the external noise, $\sigma_{e}^{2}$ , is proportional to the square of the contrast noise mask; that is,

(5) $$\sigma_{e}^{2}=w_{m}C_{m}^{2}\,$$

where *w*_m_ is a scalar constant that determines the amount of contribution of the noise mask to the variance of the response. Pooling the effects of these two noise sources, the variance of the response distribution in each detector is

(6) $$\sigma_{r}^{2}=\left(\sigma_{a}^{2}+\sigma_{e}^{2}\right)\,$$

In the context of our experiment, the observer compared the response to the stimuli in both intervals at the three possible target locations. The observer can detect the target if the difference between the response to the target + mask, *R*_j,__t+__m_, and that to the mask alone, *R*_j,m_, is greater in at least one channel than is the limitation imposed by the noise. In practice, we need to consider only the mechanism that produces the greatest response difference between the target + mask and the mask alone conditions. Thus, we can drop the subscript j for this study. That is, the decision variable *d*′ is,

(7) $$d^{\prime }=\left(R_{m+t}-R_{m}\right)/{\left(2\sigma_{r}^{2}\right)}^{1/2}\,$$

The threshold is defined when *d*′ reaches unity.

**FIGURE 4:**
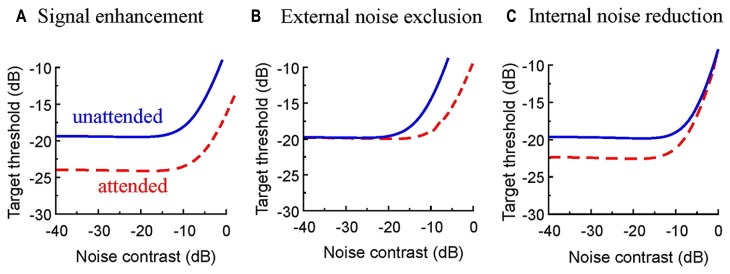
**Performance signatures in threshold contrast versus external noise contrast (TvC) functions. (A)** If the TvC functions are a vertically shifted copy of each other, that is, the same target would have different thresholds in the attended and unattended conditions, this suggests that the participant has a different sensitivity to the target in the two conditions. Hence, the effect of attention is to enhance the sensitivity [Se in Eq. (1)’] to the target in our model. **(B)** Suppose that the TvC functions for the attended condition is a rightward-shifted copy of the unattended condition. It means that the same external noise level can have different effects on target detection in the attended and unattended conditions. This suggests that attention allows the participants to exclude noise in the stimuli more easily. This corresponds to a reduction of the contribution from the external noise [*w*_m_ in Eq. (5)] in our model. **(C)** If the TvC functions showed a vertical shift at low noise contrast, but merged at high contrasts, the effect of attention is to reduce the internal noise. This corresponds to a reduction of the internal noise parameter [$\sigma_{a}^{2}$ in Eq. (6)] in our model.

**Table 1** shows the parameter of the model. To reduce the mathematical redundancy in the model, we fixed the sensitivity to the target, Se_t_, for the valid cue condition to be 100 and the size of the internal noise, $\sigma_{a}^{2}$ to be 1. As shown in the Results section, the TvC functions for the invalid conditions are vertically shifted copies of the valid condition on log–log coordinates. As shown in **Figure 4A**, such vertical shift of TvC functions can be achieved by changing the sensitivity to the target, Se_t_. Hence, our data suggest that the sensitivity to the target to be different for the valid and invalid cue conditions. This result is consistent with the models proposed by Reynolds and Heeger (2009), which suggested that spatial attention can operate in the early visual areas by affecting the attention field, and by Lu and Dosher (1998), which suggested that spatial cue enhances the target signal.

**Table 1:** The estimated parameters of the model.

	Conditions		
	Valid	Same-object	Different-object
Se_m_	2.47	2.47	2.47
Se_t_	100^*^	93.99	93.99
Si_t_	308.75	308.75	308.75
*z*	1.62	1.62	1.62
*w*_m_	5.71	5.71	11.48
σ_a_^2^	1^*^	1^*^	1^*^
*p*	3.11	3.11	3.11
*q*	2^*^	2^*^	2^*^

* Fixed value, not a free parameter.

The TvC function for the different-object condition shifted to the left from that of the same-object condition. Such horizontal shift can be implemented a change in the relative contribution of the external noise *w*_m_ (**Figure 4B**). Thus, our result suggests that the contribution of the external noise to the response variance, *w*_m_, is different in the same-object and the different-object conditions. Notice that in the valid condition, the target and the cue were also presented within the boundary of the same object. Therefore, we constrained all parameters to be the same across conditions except for sensitivity to the target, Se_t_, and the contribution of the external noise, *w*_m_. This model fits the data well; the root of mean squared error (RMSE) was 0.27. This model explains 98.61% of all variance in the averaged data.

To further validate our interpretation of the data, we tried various constraints to the model. If we constrained the sensitivity to the target, Se_t_, to be the same for all conditions, the sum of squared error (SSE) of the model increased significantly [*F*(1,12) = 73.82, *p* < 0.0001] even when we took the number of free parameters into account. Similarly, constraining the contribution of the external noise, *w*_m_, to be the same for both invalid conditions significantly increased the SSE [*F*(1,12) = 16.63, *p* < 0.05]. Therefore, the change of sensitivity to the target is necessary to explain the spatial-cueing effect while the change of the contribution of the external noise is necessary to explain the same-object advantage.

Lu and Dosher (1998) suggested a mechanism of internal noise reduction for attention. That is, the effect of the cue is to reduce the effect of the additive noise in the system. In our model, this can be implemented by changing the value of the internal noise parameter σ_a_. As shown in **Figure 4C**, such change in parameter value will cause TvC function to shift vertically in the low noise contrasts. However, the TvC function would merge together at high contrasts. We did not find such a trend in our data. Hence, our result cannot be explained by a reduction of additive internal noise. We also found that more free parameters in the model never produced a significant improvement of goodness-of-fit. Thus, no extra factors are necessary to explain our results.

## DISCUSSION

The current study systematically probed the target threshold improvement by location- and object-based attention with different noise levels using the double-rectangle method, and the results suggest that location- and object-based attention involve different mechanisms. Location-based attention operates by enhancing signal strength, whereas object-based attention operates by excluding external noise. This study is the first to demonstrate the discrepancy in the TvC functions of location- and object-based attention within a single task.

In previous studies, location- and object-based attention were examined separately by the noise-masking paradigm. Location-based attention was observed in both no-noise and high-noise conditions (Dosher and Lu, 2000; Lu and Dosher, 2000), consistent with our results. However, Han et al. (2003) found that object-based attention was also observed in both no-noise and high-noise conditions, inconsistent with our findings here. Notice that Han et al. (2003) compared the performances of tasks that required participants to attend to only *one* object versus *two* spatially separated objects. Object-based attention was indexed by higher accuracy of reporting two attributes belonging to a single object than different objects, and it was shown in both no- and high-contrast noise conditions in Han et al.’s (2003) study. It is reasonable to argue that their participants may have changed their attentional window – like a zoom lens (Eriksen and Yeh, 1985) – from “wide” in the two-object condition to “small” in the single-object condition. Accordingly, the differences between the two-object and single-object conditions not only are the number of attended objects but also the size of spatial attention (Davis et al., 2000).

This argument is supported by Liu et al. (2009) with a design identical to Han et al.’s (2003). The magnitude of the same-object advantage was modulated by the required precision of judgments: the higher the task precision, the larger the difference in performance between the two-object and the single-object conditions (Liu et al., 2009). Assuming that attentional window is wide in the two-object condition, the density of attentional resource should be low due to the reciprocal relationship between size and density of attentional distribution (Eriksen and St. James, 1986; LaBerge and Brown, 1989). The low-precision task that requires less resources can be performed equally well with less attentional resource in the two-object condition as opposed to the one-object condition – leading to reduced or no same-object advantage. The critical comparison in their study – two-object and single-object conditions – may not reflect object-based attention but rather a change in the window size of spatial attention. Indeed, the modulation pattern of “object-based” attention in Han et al.’s (2003) study is similar to the modulation pattern of location-based attention (Dosher and Lu, 2000; Lu and Dosher, 2000): both can be observed in no-noise and high-noise conditions. However, the double-rectangle method compares the same-object and different-object conditions based on an equal cue-to-target distance between the two conditions. Using the double-rectangle method, we rule out the confounding of location-based attention in the current study and find that object-based attention is observed only in high-noise conditions, indicating that external noise exclusion plays a critical role in object-based attention.

The qualitative difference between the intrinsic mechanisms of location-based and object-based attention suggests that object-based attention is not an outcome of the spreading from the location-based attention, which is a finding arguing against the well-accepted *spreading hypothesis* (e.g., Davis and Driver, 1997; Kasai and Kondo, 1997; Richard et al., 2008). Instead, we suggest that object-based attention reflects a qualitatively different kind of attentional orienting that is independent of location-based attention, rather than the modulation of an early sensory enhancement extending from location-based attention. This argument is also against the *prioritization hypothesis* proposed by Shomstein and Yantis (2002), who claimed that object-based attention reflected strategic prioritization regardless of location-based effect and that neither was it due to object-based perceptual enhancement. However, using the noise-masking paradigm, we provide evidence for the underlying mechanism of object-based attention. The current finding of the leftward-shifted copies of the TvC functions in the same-object and different-object conditions suggests that the underlying mechanism of object-based attention is to exclude external noise, an evidence of object-based perceptual enhancement.

In our experiment, the target may appear in one of the three possible locations. As a result, the participant would experience a greater uncertainty in the invalid conditions, in which the participant needed to monitor three locations, than in the valid condition, in which the participant needed to monitor just one location. Hence, one may argue that perhaps our result can be explained by uncertainty reduction (Pelli, 1985; Tyler and Chen, 2000; Chen and Tyler, 2010). Our result did show a lower threshold in the valid condition than in the invalid conditions, and in turn a vertical shift of TvC functions that is consistent with uncertainty reduction. The three-fold increase in uncertainty from the valid to the invalid cued conditions, according to Tyler and Chen (2000), translated to a 2.5 dB threshold increment. This is slightly larger than the threshold difference between the valid and the invalid cue conditions in our data (2.2 dB). Furthermore, in our experiment, there were only two location-based cueing conditions (valid and invalid). The uncertainty effect, mathematically, as discussed in the Section “Model,” can be absorbed by a change of the sensitivity parameter, Se. Thus, for practical reasons, we can consider the reduction of uncertainty as a cause of sensitivity change that accounts for the spatial cueing effect. However, uncertainty cannot explain the same-object advantage in our result. For instance, the TvC functions for the same-object and the different-object conditions were different even though the uncertainty in these two conditions was identical.

## CONCLUSION

The current study measured the thresholds in different levels of task difficulty and revealed the underlying mechanisms of location-based and object-based attention – which are difficult to evaluate from conventional RT measurements – and sheds a new light to current theories of object-based attention. Here, we overturn two widely accepted theories that object-based attention is due to the “spread” or “prioritization” of attention. In addition to revealing the underlying mechanisms of location- and object-based attention, the current finding fills the gap between previous physiological (Fink et al., 1997; He et al., 2004; Wager et al., 2004; He et al., 2008) and behavioral evidence (Shomstein and Yantis, 2004; List and Robertson, 2007; Chou and Yeh, 2008, 2011; Matsukura and Vecera, 2009) that have demonstrated the discrepancy in location-based and object-based attention by providing important convergent evidence from a novel aspect using the noise masking paradigm to the double-rectangle method.

## Conflict of Interest Statement

The authors declare that the research was conducted in the absence of any commercial or financial relationships that could be construed as a potential conflict of interest.
